# Capillary lactate as a tool for the triage nurse among patients with SIRS at emergency department presentation: a preliminary report

**DOI:** 10.1186/s13613-015-0047-y

**Published:** 2015-04-24

**Authors:** Cyril Manzon, Loïc Barrot, Guillaume Besch, Olivier Barbot, Thibaut Desmettre, Gilles Capellier, Gaël Piton

**Affiliations:** Medical Intensive Care Unit, Besançon University Hospital, Boulevard Fleming, 25030 Besançon, France; Department of Anesthesiology and Intensive Care Medicine, Besançon University Hospital, Boulevard Fleming, 25030 Besançon, France; Emergency Department, Besançon University Hospital, Boulevard Fleming, 25030 Besançon, France; Department of Epidemiology and Preventive Medicine, Faculty of Medicine, Nursing and Health Sciences, School of Public Health and Preventive Medicine, Monash University, Alfred Hospital, Commercial Rd, Melbourne, VIC 3004 Australia; Research Unit EA 3920 and SFR FED 4234, University of Franche Comté, Boulevard Fleming, 25030 Besançon, France

**Keywords:** Systemic inflammatory response syndrome, Capillary lactate, Prognosis, Triage tool

## Abstract

**Background:**

The triage nurse is involved in the early identification of the most severe patients at emergency department (ED) presentation. However, clinical criteria alone may be insufficient to identify them correctly. Measurement of capillary lactate concentration at ED presentation may help to discriminate these patients. The primary objective of this study was to identify the prognostic value of capillary lactate concentration measured by the triage nurse among patients presenting to the ED.

**Methods:**

This was a prospective observational study, performed in the ED of a university hospital. At ED presentation, capillary lactate measurement was performed by the triage nurse among patients presenting with a clinical criteria of systemic inflammatory response syndrome (SIRS). Clinical variables usually used to determine severity were collected at presentation. Twenty-eight-day mortality and MEDS score were recorded.

**Results:**

One hundred seventy-six patients with clinical SIRS presented to the ED. Median age was 72 years, and 28-day mortality was 16%. Capillary lactate at ED presentation was significantly higher among 28-day non-survivors than among survivors (5.7 mmol.L^−1^ [3.2 to 7.4] vs 2.9 mmol.L^−1^ [1.9 to 5.2], *p* = 0.003). A score based on mottling and capillary lactate concentration >3.6 mmol.L^−1^ was significantly associated with 28-day mortality (area under curve, AUC = 0.75), independently of the MEDS score (AUC = 0.79) for the prediction of 28-day mortality (AUC global model 0.87).

**Conclusions:**

A high capillary lactate concentration measured by the triage nurse among patients presenting to the ED with clinical SIRS is associated with a high risk of death. A score calculated by the triage nurse, based on mottling and capillary lactate concentration, appears to be useful for identifying the most severe patients.

## Background

The triage nurse is involved in the early identification of the most severe patients at Emergency Department (ED) presentation in order to avoid delaying treatment, to orientate patients towards the appropriate structure, and finally, to improve their prognosis [1-4]. However, clinical criteria alone can be insufficient for the triage nurse to correctly identify the most severe patients at initial presentation to the ED [5,6]. The Mortality in Emergency Department Sepsis (MEDS) score is validated for the evaluation of prognosis in patients admitted to the ED with systemic inflammatory response syndrome (SIRS) or sepsis. However, it requires a clinical diagnosis by the doctor and includes the results of biological data [7,8]. It is well established that plasma concentration of lactate is associated with the risk of death in critically ill patients [9-19], but since it requires venous or arterial blood puncture and then the time of analysis, its measurement is available only after patient care has been initiated [20]. On the contrary, capillary lactate measurement can be performed by the nurse immediately at presentation and may help to stratify patient according to their severity [21]. In addition, it is well established that there is a good correlation between plasma and capillary lactate concentrations [20]. The primary objective of this study was to evaluate the prognostic value of capillary lactate concentration among patients presenting to the ED with a clinical criteria of SIRS. Secondary objectives were to identify variables collected by the nurse at ED presentation that were associated with 28-day mortality and to build a prognostic score integrating capillary lactate concentration.

## Methods

### Study population

Adult patients presenting to the ED between November 2008 and May 2009 in a large, regional university hospital were screened immediately by the triage nurse. Inclusion criteria were: age 18 years or more and presence clinical SIRS defined by at least two criteria among the following: temperature <36°C or >38°C, heart rate >90 bpm, and respiratory rate >20/min [21]. Exclusion criteria were: traumatic injury and voluntary intoxication.

### Data collection

As part of usual practice, the following variables were prospectively collected by the triage nurse at ED presentation: age, sex, temperature (°C), Glasgow Coma Score, respiratory rate, SpO_2_, heart rate (bpm), and noninvasive assessment of blood pressure with an adjusted device according to body weight (systolic arterial pressure (mmHg), mean arterial pressure (mmHg), and diastolic arterial pressure (mmHg)). In addition, after a short educational program for the study, nurses recorded the following additional variables: presence of mottling on lower limbs, capillary refilling time on the foot (s), clinical criteria of SIRS as previously defined, and point of care capillary lactate concentration from a finger stick (Lactate Pro®, Arkray Factory, Shiga, Japan) at the same time as capillary blood glucose measurement. Doctors were blinded from capillary lactate measurement. Therapy in the ED are the following: antibiotic use, volume of vascular filling (L), catecholamine use, oxygen flow (L/min), and hospitalization after ED admission. Length of stay (days) and the final diagnosis at the end of hospitalization were recorded. Mortality at 28 days was recorded by a phone call to the patient, their family, or their general practitioner. MEDS score at ED admission was calculated [8].

### Statistical analysis

Quantitative variables are presented as median [interquartile range, IQR]. Qualitative variables are presented as number (percentage). Comparisons between qualitative variables were assessed using Fisher’s exact test or the chi-square test as appropriate. Comparisons between quantitative variables were assessed using the Wilcoxon test. A *p* value of <0.05 was considered statistically significant. A subgroup analysis was performed in the subgroup of patients presenting to the ED without hypotension. Receiver operating characteristic (ROC) curve analysis was performed to calculate the optimal cutoff values for capillary refilling time and capillary lactates to detect mortality. Because of the limited number of patients in comparison to the number of variables studied, we decided to focus on the prognostic value of mottling, capillary refilling time, and capillary lactates. Multiple logistic regression analysis was performed to evaluate whether these three variables were independently associated with 28-day mortality. Results are presented as odds ratios (OR) with 95% confidence interval (CI). A score was constructed, including the presence or absence of mottling, and two classes of capillary lactates, according to the threshold previously identified by the ROC curve analysis. Survival after ED presentation according to this score was estimated using the Kaplan-Meier analysis. All statistical analyses were performed using SAS version 9.4 (SAS Institute Inc., Cary, NC, USA).

### Patient consent

The study was approved by the ethics committee of Besançon University Hospital. Written informed consent was obtained from all patients before inclusion in the study. In the most severe patients who were unable to provide consent, written consent was obtained from the patient’s family.

## Results

### Study population

One hundred seventy-six patients with clinical criteria of SIRS at ED presentation were included in the study. The characteristics of the patients at ED presentation and the description of SIRS criteria are shown in Tables 1 and 2, respectively. Eighteen patients (10%) presented to the ED with systolic arterial pressure <90 mmHg and/or mean arterial pressure <65 mmHg. Mortality at 28 days was 16% (29/176). Capillary lactate concentration was available in 175 out of 176 patients.

**Table 1:** **Baseline characteristics of the study population at emergency department presentation**

**Variable**	***n*** **= 176**
Age (years)	72 [56 to 82]
Sex male	89 (51)
Temperature	37.3 [36.4 to 38.3]
Glasgow Coma Score	15 [15]
Ventilatory rate (bpm)	25 [22 to 30]
SpO_2_ (%)	96 [93 to 98]
Heart rate (bpm)	106 [93 to 117]
Systolic arterial pressure (mmHg)	134 [111 to 159]
Systolic arterial pressure <90 mmHg	18 (10)
Mean arterial pressure (mmHg)	92 ([77 to 108])
Diastolic arterial pressure (mmHg)	72 ([61 to 85])
Capillary refilling time (s)	1 ([1])
Presence of mottling	24 (14)
Capillary lactates (mmol.L^−1^)^a^	3.3 [2.0 to 5.8]
Length of hospitalization (days)	7 [2-13]
28-day mortality	29 (16)
MEDS score	6 [5-11]
Therapy in the ED after admission	
Antibiotic therapy	91 (52)
Volume vascular filling (L)	0.5 [0.5 to 1.5]
Catecholamine use	9 (5)
Oxygen flow (L/min)	0 [0 to 3]
Hospitalization after ED admission	150 (85)
Final diagnosis	
Pneumonia	63 (36)
Digestive disease	29 (15)
Cardiovascular disease	22 (13)
Neurological disease	15 (9)
Urinary disease	10 (6)
Cardiac arrest	2 (1)
Other diagnosis	35 (20)

Numbers are *n* (%), median [interquartile range]. ^a^Available in 175 out of 176 patients.

**Table 2:** **Characteristics of the systemic inflammatory response syndrome**

**Variable**	***n*** **= 176**
SIRS criteria	
Temperature <36°C or >38°C	92 (53)
Heart rate >90 bpm	146 (83)
Ventilatory rate >20	155 (88)
Number of SIRS criteria	
2 out of 3	135 (77)
3 out of 3	41 (23)

Numbers are *n* (%); SIRS, systemic inflammatory response syndrome.

### Univariate analysis

Univariate analysis of the relation between variables recorded by the triage nurse at ED presentation and 28-day mortality is presented in Table 3. Non-survivors at 28 days were older; had lower admission temperature, Glasgow coma score, systolic arterial pressure, mean arterial pressure, diastolic arterial pressure, and SpO_2_; more frequently had presence of mottling; had a longer capillary refilling time; and had a higher capillary lactate concentration than 28-day survivors (all *p* < 0.05). There was a positive correlation between the capillary lactate concentration measured at presentation and the volume of vascular filling administered in the ED (*R* = 0.26, *p* = 0.0005). ROC curve analysis identified a threshold of 2 s for capillary refilling time and 3.6 mmol.L^−1^ for capillary lactate concentration as best predicting 28-day mortality (area under curve (AUC) 0.66 for each).

**Table 3:** **Univariate analysis of the variables at ED presentation according to 28-day mortality**

	**Survivors at 28 days**	**Non-survivors at 28 days**	***p***
	***n*** **= 147**	***n*** **= 29**	
Age (years)	66 [51 to 81]	79 [69 to 83]	0.004
Sex male	70 (48)	19 (66)	0.10
Temperature (°C)	37.4 [36.5 to 38.4]	36.9 [35.6 to 37.8]	0.046
Glasgow Coma Score	15 [15]	14 [11 to 15]	<0.0001
Heart Rate (bpm)	105 [95 to 116]	106 [91 to 120]	0.85
Systolic arterial pressure (mmHg)	137 [113 to 160]	113 [88 to 150]	0.04
Systolic blood pressure <90 mmHg	10 (7)	8 (28)	0.003
Mean arterial pressure (mmHg)	96 [80 to 109]	86 [69 to 98]	0.03
Diastolic arterial pressure (mmHg)	74 [62 to 86]	63 [56 to 79]	0.04
Ventilatory rate (bpm)	25 [22 to 30]	29 [24 to 31]	0.10
SpO_2_ (%)	96 [94 to 98]	95 [90 to 96]	0.02
Capillary refilling time (s)	1 [1 to 1]	1 [1 to 3]	0.0001
Presence of mottling	11 (7)	13 (45)	<0.0001
Capillary lactates	2.9 [1.9 to 5.2]	5.7 [3.2 to 7.4]	0.003

Numbers are *n* (%), median [interquartile range].

### Logistic regression analysis

The results of logistic regression analysis of mottling, capillary refilling time, and capillary lactate concentration to predict 28-day mortality are shown in Table 4.

**Table 4:** **Logistic regression analysis to predict 28-day mortality in patients presenting to the ED with clinical SIRS**

**Variables**	**Simple logistic regression**		**Multiple logistic regression**	
	**OR**	***P***	**OR**	***P***
Capillary refilling time		<0.0001		0.013
≤2 s	1		1	
>2 s	18.9 [5.4 to 66.7]		6.6 [1.5 to 29.4]	
Mottling		<0.0001		0.014
Absence	1		1	
Presence	10 [3.9 to 26.3]		4.4 [1.35 to 14.5]	
Capillary lactates		0.003		0.019
≤3.6 mmol/L	1		1	
>3.6 mmol/L	3.8 [1.6 to 9.1]		3.2 [1.2 to 8.4]	

OR, odds ratio [confidence interval 95%].

By simple logistic regression, capillary refilling time >2 s, presence of mottling, and capillary lactate concentration >3.6 mmol.L^−1^ were all significantly associated with 28-day mortality (OR [CI 95%] = 18.9 [5.4 to 66.7], OR [CI 95%] = 10 [3.9 to 26.3], and OR [CI 95%] = 3.8 [1.6 to 9.1], respectively). By multiple logistic regression, capillary refilling time >2 s, presence of mottling, and capillary lactates >3.6 mmol.L^−1^ were independently associated with 28-day mortality (OR = 6.6 [1.5 to 29.4], OR = 4.4 [1.35 to 14.5], and OR = 3.2 [1.2 to 8.4], respectively).

### Score based on mottling and capillary lactates

Since capillary lactate concentration and mottling at presentation were independently associated with 28-day mortality, a score based on the presence or absence of mottling, and capillary lactate concentration ≤3.6 mmol.L^−1^ or >3.6 mmol.L^−1^ was built (Table 5). Mortality at 28 days was 6%, 19%, and 67% among patients with score of 0, 1, or 2, respectively (*p* < 0.0001). Among the 157 patients presenting to ED without hypotension (i.e., systolic arterial pressure ≥90 mmHg and mean arterial pressure ≥65 mmHg), 28-day mortality was 6%, 17%, and 56% for a score of 0, 1, or 2, respectively (*p* = 0.0005).

**Table 5:** **Nurse hypoperfusion score in patients with clinical SIRS at ED presentation**

**Definition of score**	**28-day mortality**				
	**Overall SIRS**		***p***	**SIRS without hypotension**	***p***
Absence of mottling and capillary lactates ≤3.6 mmol/L	0 point	5/85 (6)	<0.0001	5/84 (6)	0.0005
Presence of mottling or capillary lactates >3.6 mmol/L	1 point	14/75 (19)		11/64 (17)	
Presence of mottling and capillary lactates >3.6 mmol/L	2 points	10/15 (67)		5/9 (56)	

**Figure 1:**
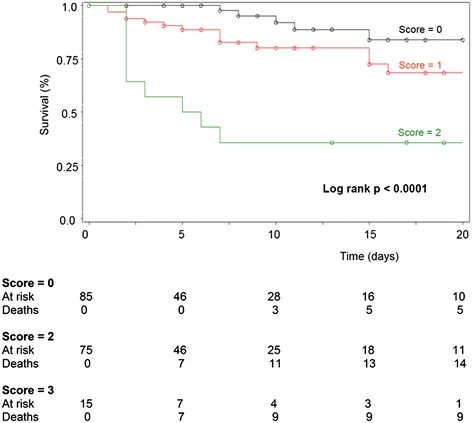
Survival after ED presentation based on mottling and capillary lactates estimated using the Kaplan-Meier method. Kaplan-Meier survival curve of patients with clinical SIRS at emergency department presentation according to the nurse hypoperfusion score. Vertical axis represents estimated probability of survival. Horizontal axis represents time in days after emergency department admission. Censored observations, corresponding to patient survivors that were lost of follow-up, are represented by circles.

Survival after ED presentation according to this score was estimated using the Kaplan-Meier method (Figure 1, log-rank *p* < 0.0001). The characteristics of the patients at ED presentation according to the score are presented in Table 6. Care intensity in the ED according to the score is presented in Table 7. The higher the score, the higher the volume of vascular filling, the higher the oxygen flow, and the more frequent the catecholamine use. The AUC for the score and for the MEDS score were 0.75 and 0.79, respectively. Multiple logistic regression analysis showed that the two scores were independent for the prediction of 28-day mortality (AUC of the global model = 0.87).

**Table 6:** **Characteristics of the patients at ED presentation according to the nurse hypoperfusion score**

	**Score = 0**	**Score = 1**	**Score = 2**
***n***	**85**	**75**	**15**
Age (years)	75 [56–83]	66 [53 to 79]	79 [61 to 85]
Temperature (°C)	37.3 [36.7 to 38.3]	37.4 [36.4 to 38.3]	36.5 [35.3 to 38.2]
Glasgow Coma Score	15 [15 to 15]	15 [14 to 15]	14 [8 to 15]
Heart Rate (bpm)	102 [95 to 112]	108 [94 to 117]	95 [76 to 120]
Systolic arterial pressure (mmHg)	142 [121 to 163]	130 [104 to 156]	110 [85 to 143]
Mean arterial pressure (mmHg)	96 [85 to 112]	92 [73 to 106]	73 [60 to 103]
Diastolic arterial pressure (mmHg)	75 [64 to 88]	71 [58 to 83]	59 [46 to 81]
Ventilatory rate (bpm)	25 [22 to 29]	25 [22 to 30]	30 [21 to 34]
SpO_2_ (%)	96 [94 to 98]	96 [94 to 98]	96 [91 to 98]
Capillary refilling time (s)	1 [1 to 1]	1 [1 to 1]	3 [1 to 3]

Numbers are median [interquartile range].

**Table 7:** **Care intensity in the ED according to the nurse hypoperfusion score calculated at ED presentation**

	**Score = 0**	**Score = 1**	**Score = 2**	***p***
***n***	**85**	**75**	**15**	
Oxygen flow (L/min)	0 [0 to 2]	2 [0 to 3]	3 [1 to 6]	0.006
Vascular filling (L)	0.5 [0.5 to 1]	0.9 [0.5 to 1.5]	1.5 [0.5 to 2.5]	0.0002
Catecholamine use	0 (0)	5 (7)	4 (27)	<0.0001
Antibiotic therapy	38 (45)	41 (55)	11 (73)	0.09

Numbers are median [interquartile range], *n* (%).

## Discussion

The main result of this study is that a high capillary lactate concentration measured by the triage nurse at ED presentation among patients with clinical criteria of SIRS is associated with a high risk of death. This study reinforces the role of the triage nurse.

To the best of our knowledge, this is the largest study evaluating the prognostic value of capillary lactate concentration at ED presentation in terms of the number of patients included. Only one study has specifically studied the prognostic value of capillary lactate at ED admission. Seoane et al., in their prospective study of 79 patients with critical illness, identified that a capillary lactate concentration of >2.35 mmol.L^−1^ was associated with mortality [20]. In the present study, we identified a cutoff of 3.6 mmol.L^−1^ that best predicted 28-day mortality (OR = 3.8 [1.6 to .1], *p* = 0.003). This cutoff is close to the cutoff of 4 mmol.L^−1^ recommended to identify patients with severe sepsis [2].

Measuring capillary lactate concentration may help to identify patients with altered microcirculation. Indeed, in our study, 90% of patients presented to the ED without hypotension but with increased capillary lactate concentration. Among the variables collected by the triage nurse, we found that the capillary refilling time was associated with 28-day mortality, with an optimal cutoff of 2 s (OR = 18.9 [5.4 to 66.7], *p* < 0.0001). However, only 8% of patients had a capillary refilling time >2 s. Intuitively, one could imagine that an elevated capillary refilling time provides the same information as the presence of mottling or increased lactate concentration. However, interestingly, presence of mottling, capillary lactate concentration >3.6 mmol.L^−1^, and capillary refilling time >2 s were independent of each other for the identification of 28-day non-survivors.

A score integrating capillary lactate concentration with a cutoff of 3.6 mmol.L^−1^, and presence or absence of mottling, appears to be a useful tool for determining severity of disease in these patients. Mortality at 28 days was 6%, 19%, and 67% among patients with a score 0, 1, or 2, respectively (*p* < 0.0001). This nurse hypoperfusion score presents several advantages.

Firstly, it can be calculated by a nurse and not necessarily by a doctor. Secondly, it can be obtained immediately at patient presentation, avoiding the time delay engendered by admission procedures, arterial or venous blood puncture, and the time required for laboratory analyses. Indeed, it has been demonstrated that the time required for capillary lactate assessment was significantly shorter than that needed for arterial or venous lactate assessment [20]. Thirdly, this score does not take into account variables that are clearly associated with patient’s severity. Indeed, coma, hypotension, or acute respiratory failure are clinically obvious, and these patients are immediately oriented towards a medical doctor. On the contrary, our score takes into account variables that could reflect occult tissue hypoperfusion, even in patients with normal vital signs. Indeed, the score was strongly associated with 28-day mortality, even after restricting the analysis to patients presenting to the ED without hypotension (Table 5). Analysis of the patient’s characteristics at ED presentation according to the three classes of the score showed that the median systolic or mean arterial pressure, the median SaO_2_, and the median Glasgow Coma Score were all within normal range (Table 6). Since all the patients had clinical criteria of SIRS, it can be observed that median heart and ventilatory rates were increased. Furthermore, a recent study showed that some patients do not have increased plasma lactate concentration despite an unfavorable outcome including vasopressor-dependant septic shock [22]. There is thus a likely benefit to be gained from using such a composite score that integrates both capillary lactate dosage and clinical signs of hypoperfusion. Although promising, these results are preliminary, in view of the sample size. The utility of such a score needs to be evaluated prospectively in a large cohort of patients.

The MEDS score is the most validated prognostic score among patients admitted to the ED with sepsis or SIRS [7,8,23]. Its calculation requires the intervention of a medical doctor for the evaluation of fatal disease and diagnoses of pneumonia and septic shock, and it is also necessary to wait for the results of biological analyses for leucocyte and platelet counts. Therefore, the MEDS score cannot be calculated by a nurse and cannot be calculated immediately at patient presentation. Two findings of the present study deserve to be underlined. Firstly, the AUC for our microcirculatory dysfunction score was similar to the AUC of the MEDS score for predicting 28-day mortality (0.75 and 0.79, respectively), suggesting that our score is efficient for discriminating the most severe patients. Secondly, multiple logistic regression analysis showed that the two scores were independent of each other for the prediction of 28-day mortality, suggesting that they give different information regarding prognosis (AUC of the global model 0.87). Finally, these two scores could be complementary, with one performed by the triage nurse at patient presentation and the second calculated by the doctor after admission and evaluation of the patient.

Improving patient triage is a matter of importance, in particular, in case of high affluence to the ED, a situation where there is a risk of underestimating severity due to lack of time and means. In this setting, reinforcing the role and the performance of the triage nurse may help the medical doctor to allocate the appropriate care to the patients. In the present study, the nurse did not inform the medical doctor of capillary lactate concentration. However, the score calculated by the triage nurse at presentation was associated with care intensity in the ED. It remains to be evaluated whether taking the results of the nurse hypoperfusion score into account might improve the patient’s prognosis. For example, patients presenting to the ED with a score of 0, with an expected 28-day mortality risk of 6%, could be oriented to usual care. On the contrary, those with a score of 1 or 2, with an expected 28-day mortality risk ranging between 19% and 67%, should be immediately evaluated by an intensive care specialist and oriented towards an ICU. By avoiding delay in the first precious hours of care, for example, the golden hour for initiation of antibiotic therapy, such a strategy could considerably improve the prognosis of patients presenting to the ED.

This study has several limitations. First, this was a monocentric study. It is necessary to evaluate the interest and limits of the nurse hypoperfusion score in a large multicentric study, and in particular, to compare it with the MEDS score. Second, during the study period, consecutive patients presenting to the ED were not all included. Indeed, triage nurses who did not participate to the short educational program did not participate to the study. Third, arterial or venous lactate concentration was not measured in all patients, and therefore, a correlation between capillary and plasma lactate concentration was not available. However, a recent study has found a good correlation between capillary and plasma lactate concentrations [20].

## Conclusions

In conclusion, a high capillary lactate concentration measured by the triage nurse among patients presenting to the ED is associated with a high risk of death. A score based on mottling and capillary lactate concentration, calculated by the triage nurse, appears to be useful to identify the most severe patients.

## Abbreviation

ED: emergency department
